# The impacts of *H. pylori* virulence factors on the development of gastroduodenal diseases

**DOI:** 10.1186/s12929-018-0466-9

**Published:** 2018-09-11

**Authors:** Wei-Lun Chang, Yi-Chun Yeh, Bor-Shyang Sheu

**Affiliations:** 1 Department of Internal Medicine, National Cheng Kung University Hospital, College of Medicine, National Cheng Kung University, 138 Sheng Li Road, Tainan, Taiwan; 2 Department of Internal Medicine, Tainan Hospital, Ministry of Health and Welfare, Tainan, Taiwan

**Keywords:** Virulence factors, *Helicobacter pylori*, Gastric adenocarcinoma, Gastric B cell lymphoma, Peptic ulcer disease

## Abstract

Although most *H. pylori* infectors are asymptomatic, some may develop serious disease, such as gastric adenocarcinoma, gastric high-grade B cell lymphoma and peptic ulcer disease. Epidemiological and basic studies have provided evidence that infection with *H. pylori* carrying specific virulence factors can lead to more severe outcome. The virulence factors that are associated with gastric adenocarcinoma development include the presence, expression intensity and types of cytotoxin-associated gene A (CagA, especially EPIYA-D type and multiple copies of EPIYA-C) and type IV secretion system (CagL polymorphism) responsible for its translocation into the host cells, the genotypes of vacuolating cytotoxin A (*vacA*, s1/i1/m1 type), and expression intensity of blood group antigen binding adhesin (BabA, low-producer or chimeric with BabB). The presence of CagA is also related to gastric high-grade B cell lymphoma occurrence. Peptic ulcer disease is closely associated with *cagA*-genopositive, *vacA* s1/m1 genotype, *babA2*-genopositive (encodes BabA protein), presence of duodenal ulcer promoting gene cluster (*dupA* cluster) and induced by contact with epithelium gene A1 (*iceA1*), and expression status of outer inflammatory protein (OipA). The prevalence of these virulence factors is diverse among *H. pylori* isolated from different geographic areas and ethnic groups, which may explain the differences in disease incidences. For example, in East Asia where gastric cancer incidence is highest worldwide, almost all *H. pylori* isolates were *cagA* genopositive, *vacA* s1/i1/m1 and BabA-expressing. Therefore, selection of appropriate virulence markers and testing methods are important when using them to determine risk of diseases. This review summarizes the evidences of *H. pylori* virulence factors in relation with gastroduodenal diseases and discusses the geographic differences and appropriate methods of analyzing these virulence markers.

## Background

*H. pylori* infection is highly prevalent affecting half of the world’s population. Once infected, *H. pylori* can be a lifelong infection in the host unless eradicated. Nevertheless, about 85% of the infected patients only have mild asymptomatic gastritis, while 15% of patients can develop to have peptic ulcer disease (PUD), and less than 1% can develop to have gastric cancer [1].

The diverse clinical presentation of *H. pylori* infection is a result of interaction between bacterial virulence (e.g. CagA, VacA, BabA), host genetic (e.g. IL-1β, IL-10, TNF-α), and environmental factors (e.g. diet, smoke). The virulence factors of *H. pylori* can be categorized to be related with 3 major pathogenic processes, including colonization, immune escape and disease induction (Table 1). The virulence factors responsible for establishing colonization include urease, flagella, chemotaxis system, and adhesins [2, 3]. Knocking out the urease, flagella or chemotaxis genes all leads to a failure of *H. pylori* to establish colonization [3]. With increasing antibiotic resistance, these virulence factors provide alternative drug or vaccine targets for *H. pylori* eradication and prevention [4]. The virulence factors responsible for immune escape help *H. pylori* escape from host immune clearance and allow its persistence in the human stomach [5]. This review focused on the virulence factors causing more serious clinical outcomes.

**Table 1:** The 3 categories of *H. pylori* virulence factors and their functions

The three categories of virulence factors	Biological functions or associated diseases
**Colonization**	**Biological functions**
Urease	Neutralize gastric acid
Flagella Chemotaxis system	Bacterial movement to epithelial surface & deep gland
Adhesins	Adherence to gastric epithelial cells
• BabA	
• SabA	
• Lewis antigens	
• OipA	
**Immune escape**	**Biological functions**
LPS & Flagella	Low immunogenicity Molecular mimicry Induce anti-inflammatory response
CagA & T4SS	Suppress phagocytosis Decrease antimicrobial peptide Induce tolerogenic dendritic cell Block effector T cell response
VacA	Suppress phagocytosis Induce tolerogenic dendritic cell Block effector T cell response
Gamma-glutamyl transpeptidase	Induce tolerogenic dendritic cell Block effector T cell response
Cholesterol-α-glucosyltransferase	Suppress phagocytosis
Catalase Superoxide dismutase	Suppress and ROS & NO
Arginase	Suppress ROS & NO Block effector T cell response
**Disease induction**	**Associated diseases**
CagA & T4SS	Gastric adenocarcinoma, MALToma & PUD
VacA	Gastric adenocarcinoma & PUD
BabA	Gastric adenocarcinoma & PUD
HtrA	Gastric adenocarcinoma
DupA	Duodenal ulcer
IceA	PUD
OipA	PUD

*LPS* Lipopolysaccharide, *T4SS* type IV secretion system, *ROS* reactive oxygen species, *NO* nitric oxide, *PUD* peptic ulcer disease

## Virulence factors associated with gastric adenocarcinoma

Gastric adenocarcinoma is the most deadly disease cause by *H. pylori*. The virulence factors that are associated with development of gastric adenocarcinoma are summarized in Table 2.

**Table 2:** The *H. pylori* virulence factors associated with gastric adenocarcinoma

Region	Virulence factor (High risk marker)	Odds ratio	Analytic method	Reference
East Asia	CagL Y58E59 (vs. non-Y58E59)	4.6	DNA sequencing	[25]
	Bab AB BA recombination (vs. others)	6.2	PCR	[44]
Middle Asia	*vacA* i1 (vs. i2)	10.9	PCR	[34]
Middle East	*vacA* s1 (vs. s2)	4.0	PCR	[36]
	*vacA* m1 (vs. m2)	2.5	PCR	[36]
	*vacA* s1/m1 (vs. s2/m2)	5.3	PCR	[36]
	*vacA* i1 (vs. i2)	15.0	PCR	[36]
Asia	EPIYA-D motif of CagA (vs. EPIYA-C)	1.9	DNA sequencing	[30]
Western countries	Positive serum anti-CagA Ab	2.0	Serum ELISA	[19]
	*cagA*+ (vs. *cagA*-)	2.4	PCR or Immunoblot	[20]
	≥ 2 copies of EPIYA-C motifs of CagA (vs. 1 copy)	3.3	DNA sequencing	[30]
	Western EPIYA-B motif of CagA (vs. EPIYT-B)	3.0	DNA sequencing	[29]
	*vacA* s1 (vs. s2)	5.3	PCR	[20]
	*vacA* m1 (vs. m2)	2.5	PCR	[20]
	*vacA* s1/m1 (vs. s2/m2)	4.4	PCR	[20]
	BabA-L (vs. BabA-negative)	33.9	Immunoblot	[46]
	BabA-H (vs. BabA-negative)	18.2	Immunoblot	[46]
	*oipA* “on” (vs. *oipA* “off”)	2.4	DNA sequencing or immunoblot	[74]

*PCR* Polymerase chain reaction

### Cytotoxin-associated gene a (CagA) & type IV secretion system (T4SS)

CagA is a well-recognized oncoprotein which is injected into host cells via a pilus structure called type IV secretion system (T4SS) [6]. Successful pilus formation and CagA translocation relies on the binding between CagL on the tip of T4SS and integrin α5β1 receptor on the host cell [7]. The gene locus that encodes CagA and the T4SS is called *cag* pathogenicity island (*cag* PAI). After being injected into host cells, CagA alters intracellular signal transduction pathways that facilitates malignant transformation of gastric epithelial cells or activates Lgr5-positive stem cells [8, 9]. Importantly, transgenic mice overexpressing phosphorylation-competent CagA developed gastrointestinal adenocarcinoma, myeloid leukemia and B cell lymphoma, while phosphorylation-resistant CagA could not confer the same pathological changes [10]. These data provided direct evidence that CagA is an oncoprotein and the need of phosphorylation in conferring oncogenesis.

In addition to the above cellular changes, CagA and T4SS also increase gastric inflammation via NFκB signaling and increased IL-8 secretion [11, 12], which predispose to genetic instability and carcinogenesis. CagA can also cause epigenetic changes, such as DNA promoter or histone hypermethylation, which in turn leads to downregulation of tumor suppressor genes (e.g. MGMT) or microRNAs (e.g. let-7) [13, 14]. Alternatively, CagA can attenuate tumor suppressing microRNA expression (e.g. miR-320a, miR-4496) via unknown mechanisms [15, 16]. Therefore, CagA and T4SS can contribute to gastric carcinogenesis via multiple mechanisms.

In concordance with the above-mentioned in vitro and in vivo evidences, several epidemiologic studies also support infection with CagA-positive *H. pylori* can increase the gastric cancer risk, especially for the non-cardiac location. Most of these studies just tested the serum antibodies against CagA protein to define the status of a CagA-positive *H. pylori* infection [17–19]. Meta-analyses of case-control studies showed CagA-seropositive is associated with 2-fold risk of distal gastric cancer among *H. pylori* infectors [19], while *cagA*-genopositive is associated with 2.1–2.4 fold risk of gastric cancer [20]. A cohort study with long-term follow-up also demonstrated that infection with *cagA*-genopositive strain was associated with greater risk of progression of gastric precancerous lesion (OR = 2.28). However, in East Asia, where nearly all *H. pylori* strains possess *cagA* gene [21], presence of serum anti-CagA antibody or *cagA* gene may not be sensitive enough [22], and CagA subtyping is suggested to identify high risk infectors (Table 2).

Accordingly, the risk of gastric cancer in CagA-positive *H. pylori* infector can be further stratified by CagA expression level [23, 24], the amount of translocation into host cell [25, 26] and its biological activity [27–29]. CagA expression level is higher with the presence of genetic AATAAGATA motif upstream of the translation-starting site, which was associated with greater risk of advanced gastric precancerous lesion [23, 24]. The amount of CagA translocation is greater in strains harboring an amino acid sequence polymorphism (Y58E59) in the CagL of T4SS, which increases its binding affinity with integrin receptor α5β1 on the gastric epithelial cell [26]. Accordingly, gastric cancer risk was increased by 4.6-fold in patients infected by CagL-Y58E59 strain compared with those infected by non-Y58E59 strain [25].

The biological activity of CagA protein is determined by the types and numbers of the EPIYA motifs on its C-terminal region. There are four types of EPIYA motifs based on their distinct conserved flanking sequences, namely EPIYA-A, -B, -C and -D. *H. pylori* isolates from East Asia where gastric cancer incidence is highest often contains EPIYA A-B-D motif, whereas isolates from Western countries often contains EPIYA A-B-C motif. The A-B-D motif has stronger Src homology 2 phosphatase (SHP-2) binding affinity than the A-B-C motif [27]. A meta-analysis showed 1 EPIYA-D motif was associated with 1.91-fold gastric cancer risk in Asia, compared with 1 EPIYA-C motif [30]. In Western countries, strains harboring multiple EPIYA-C motifs (A-B-C-C or A-B-C-C-C) have higher phosphorylation capacity, SHP-2 binding affinity, and confer higher gastric cancer risk (OR = 3.28) compared with only 1 EPIYA-C motif [30]. Notably, a higher CagA phosphorylation ability was associated with increased risk of gastric precancerous lesions in Taiwan [31]. In addition, amino acid sequence polymorphism within the Western EPIYA-B motif also influences CagA activity, as strains harboring EPIYT-B motif have attenuated ability of inducing hummingbird phenotype and IL-8 in gastric epithelial cells and confer less gastric cancer risk than strains harboring EPIYA-B motifs [29]. Interestingly, EPIYT-B motif was associated with higher duodenal ulcer risk [29].

### Vacuolating cytotoxin a (VacA)

VacA was named for its ability to induce vacuole formation in eukaryotic cells. The difference in vacuolating abilities are determined by the variations in the three regions of the *vacA* gene — the signal (s1 and s2), intermediate (i1 and i2) and middle regions (m1 and m2). A combination of different sequences in the 3 regions leads to multiple alleles and determines the vacuolating activity. Vacuolating activity is high in s1/m1 genotypes, intermediate in s1/m2 genotypes, and absent in s2/m2 genotypes [32]. In clinical isolates, only s1/m2 strain varies in i-type; s1/m1 and s2/m2 strains are exclusively i1 and i2, respectively [33]. The i-type determines vacuolating activity among the s1/m2 strains [33]. Even though the physiologic role of vacuolation is unclear, higher vacuolation activity was linked with more severe clinical outcomes of the infection.

Meta-analysis showed individuals infected with *H. pylori* harboring *vacA* s1 or m1 has an increased risk of gastric cancer in Western populations (OR = 2.50–5.32, Table 2) [20]. Moreover, *vacA* i1 type *H. pylori* infection is associated with higher gastric cancer risk in the Middle Asia and Middle East area (OR = 10.9–15.0) [34]. However, similar to CagA, the high prevalence of *vacA* s1/i1/m1 genotype in the East Asian and Southeast Asian population dampens its application as a disease determinant in these areas [35].

Interestingly, the s1/i1/m1 type of *vacA* is often linked with genopositive *cagA* [36]. Therefore, neither of the virulence markers can be considered an independent factor for disease outcome [37]. In fact, when multiple virulence factors are present, the risk of severe clinical outcome is greater. For example, in a long-term follow-up cohort, infection with strains that are simultaneously *cagA*-genopositive and *vacA* s1/m1 was associated 4.8-fold risk of progression of gastric precancerous lesions compared to those infected with *cagA*-negative/*vacA* s2/m2 strains, which was higher than each of the virulence factors considered alone (OR = 2.28–3.38) [38].

### Blood group antigen binding adhesin (BabA)

BabA encoded by *babA2* gene is a major adhesin on the outer membrane of *H. pylori*, which recognizes Lewis b (Le^b^) blood group antigens on the host cells and determines *H. pylori* colonization density [39, 40]. Two other paralogs of BabA had been found — the BabB and BabC, encoded by *babB* and *babC* gene, respectively. The sequence of the 3 *bab* genes was similar in the 5′ and 3′ region particularly between *babA* and *babB,* but the middle region was diverse and likely mediates the binding function. Thus, only BabA has Le^b^ antigen binding ability [41, 42]. The BabA protein expression is mainly regulated by the recombination between *babA* and *babB* gene, which forms chimeric proteins (BabA/B or BabB/A) [41, 43, 44]. For example, intra-genomic recombination with *babB* brings variable number of cytosine-thiamidine (CT) dinucleotide to the 5′-region of the *babA* sequence, leading to phase variation and affects the expression of BabA [43]*.* Other mechanisms that regulate BabA expression includes mutation in the coding region of the *babA2* gene, or the presence of 4 additional adenines (poly[A] nucleotides) within the − 10 to − 35 region of the *babA2* promoter, which diminishes the strength of the promoter [43].

Therefore, using a single pair of PCR primers to determine *babA2* genopositivity may not reflect the actual expression status of BabA. This may explain the conflicting results of studies exploring the correlation between *babA2* genopositivity and gastric cancer [45]. Fujimoto et al. determined BabA expression level by immunoblotting and classified *H. pylori* into BabA high producers (BabA-H) with Le^b^ binding activity, BabA low producers (BabA-L) without Le^b^ binding activity, and BabA-negative strain (*babA2*-genonegative) [46]. Notably, infection with BabA-L strains was associated with highest risk of gastric cancer, followed by infection with BabA-H and BabA-negative strains. In Western countries, infection with BabA-L and BabA-H strain are associated with 54.8-fold and 19.8-fold risk of gastric cancer compared to BabA-negative infectors. Moreover, BabA-L strain infectors had highest gastric *H. pylori* colonization density, neutrophil infiltration, and mucosal atrophy. However, since all *H. pylori* isolates from East Asia are either BabA-H or BabA-L, the categorization is not sensitive enough to risk stratify infectors in this area.

In Taiwan, we explored *babA* and *babB* recombination using multiple pairs of PCR primers. Four types of *babA* and *babB* recombination can be found — the A B genotype without recombination, AB B with *babB* inserted into *babA*, A BA with *babA* inserted into *babB*, and AB BA with both of the recombination [44]. The isolates from gastric cancer patients had a higher rate of AB BA genotype than those from non-cancer patients (40.0% vs. 9.7%, OR = 6.2, *p* < 0.05). Interestingly, isolates with AB BA genotype had lower BabA expression level than isolates with A B genotype [44]. Therefore, although *babA2*-genonegative strain was associated with lowest gastric cancer risk [46], in *babA2*-genopositive strain, a lower BabA expression level seemed to be associated higher gastric cancer risk [44, 46]. These data suggest multiple pairs of PCR primers should be used to reflect actual BabA status and determine the risk of gastric cancer, especially in East Asia where nearly 100% *H. pylori* are *babA2*-genopositive [40, 46].

Notably, *H. pylori* that simultaneously expresses multiple virulence factors is associated with an even higher risk of severe clinical outcomes. A case-control study showed Infection with strains “triple-positive” for *cagA*, *vacAs1* and *babA2* genes significantly correlates to the development of peptic ulcer (*p* < 0.0001) and adenocarcinoma (*p* = 0.014) and discriminated adverse disease outcome better than did the dual-positive (*cagA* and *vacA1*) classification [47].

### High temperature requirement a (HtrA)

*H. pylori* can secrete proteases as well as induce the expression of host proteases to cleave extracellular matrix and intercellular junctional proteins. Disruption of junctional protein is particularly important for *H. pylori* to exploit the host receptors located on the basolateral side of the cell membrane, such as integrin [48]. The serine protease and chaperone HtrA is most studied protease expressed by *H. pylori*. Intracellular HtrA acts as chaperone that refold and degrade misfolded proteins. Thus, HtrA is important for bacterial survival under stressful conditions, such as extreme temperature, pH or salt concentration [49]. All clinical *H. pylori* isolates possesses *htrA* gene and suppression of HtrA proteolytic activity is sufficient to kill *H. pylori* [50]. Therefore, HtrA is a promising target for anti-*H. pylori* therapy. In addition to the essential role in *H. pylori* survival, secreted HtrA can cleave E-cadherin and fibronectin [51]. E-cadherin cleavage disrupts cell junctions which exposes basolateral integrin receptors for binding by the *H. pylori* T4SS, as well as induces epithelial-mesenchymal transition. Since fibronectin has integrin binding motif — RGD, its proteolysis may release integrin receptors on the gastric epithelial cells to interact with *H. pylori* T4SS, and subsequently facilitate the translocation of CagA [7]. It is novel to assess whether *htrA* genetic polymorphism is associated with gastric cancer risk, especially in the high gastric cancer incidence area.

## Virulence factors associated with gastric B cell lymphoma

Previous studies showed *cagA* gene was found more frequently (*p* < 0.05) in the biopsies of gastric high-grade B cell lymphoma (76.7%, 23/30) compared to the gastritis (30.3%, 17/56) and the low-grade lymphoma of the mucosa associated lymphoid tissue (MALToma) cases (37.8%, 14/37) [52]. In addition, the prevalence of serum anti-CagA antibody was higher (*p* < 0.05) in patients with gastric diffuse large B cell lymphoma (75%, 12/16) than those with low-grade MALToma (44.8%, 13/29) and non-ulcer dyspepsia (43.1%, 22/53) [53]. These data indicates CagA is associated with development of gastric high-grade B cell lymphoma.

In vitro study showed CagA is able to be translocated into human B lymphocytes via T4SS [54]. Once in the cytoplasm, it binds to SHP-2, which stimulates B lymphocyte proliferation and inhibits apoptosis via regulation of intracellular pathways, including activation of endoplasmic reticulum kinases 1 and 2 (ERK 1 and ERK 2) and p38 MAP kinase (MAPK) and increased expression of Bcl-2 and Bcl-xL [54]. Clinical study also showed positive correlations between the expression of CagA and phospho-SHP-2 (p-SHP-2), p-ERK, p-p38 MAPK, Bcl-2 and Bcl-xL in gastric MALToma tissue [55]. Therefore, CagA may promote gastric low-grade MALToma transformation to high-grade B cell lymphoma via the above pathways.

Recently, the genomes of three *H. pylori* strains isolated from MALToma patients were sequenced. Nine genes were identified to be shared by 3 MALToma strains and absent in the reported 5 gastritis/ulcer strains [56]. Further investigations are needed to clarify the impact of these genes in gastric lymphomagenesis.

## Virulence factors associated with peptic ulcer disease (PUD)

The virulence factors that are associated with development of PUD are summarized in Table 3.

**Table 3:** The *H. pylori* virulence factors associated with peptic ulcer disease

Region	Virulence factor (High risk marker)	Odds ratio	Analytic method	Reference
East Asia	≥ 2 copies of EPIYA-C motifs of CagA increases DU risk (vs. 1 copy)	2.3	DNA sequencing	[30]
Southeast Asia	*cagA*-genopositive	2.8	PCR	[58]
	*vacA* m1 (vs. m2)	1.5	PCR	[58]
Middle East	*vacA* s1 (vs. s2)	3.1	PCR	[36]
	*vacA* m1 (vs. m2)	1.8	PCR	[36]
Asia	*dupA*-genopositive increases DU risk	1.6	PCR	[63]
Western countries	*cagA*+ (vs. *cagA*-)	1.7	PCR or Immunoblot	[20]
	*vacA* s1 (vs. s2)	1.7	PCR	[59]
	*vacA* s1/m1 (vs. s2/m2)	2.0	PCR	[20]
	*babA2*-genopositive	2.1	PCR	[45]
	BabA-L (vs. BabA-negative)	54.8	Immunoblot	[46]
	BabA-H (vs. BabA-negative)	19.8	Immunoblot	[46]
	*dupA* cluster positive increases DU risk	2.1	PCR	[66]
	*iceA1*-genopositive	1.3	PCR	[70]
	*oipA* “on” (vs. *oipA* “off”)	4.0	DNA sequencing or immunoblot	[74]

*PCR* Polymerase chain reaction

### Cytotoxin-associated gene a (CagA)

In a large meta-analysis including 44 studies and 17,374 patients from both Eastern and Western regions, CagA-seropositive was associated with a 1.69-fold risk of PUD, which was lower than its association with gastric cancer (OR = 2.44) [20]. However, due to diverse *cagA* genoprevalence in the various geographic areas, the methods used to identify high risk population for PUD should be different. In Western and Southeast Asian population, where *cagA*-genopositive rate is lower, *cagA*-genopositive is sensitive enough to identify high risk infector for PUD [57, 58]. In East Asia, where nearly all *H. pylori* strains possess *cagA* gene [21], CagA subtyping is suggested to identify high risk infectors. Accordingly, a meta-analysis showed multiple EPIYA-C motifs is associated with 2.3-fold risk of DU compared with 1 EPIYA-C motif in Asian population [30].

### Vacuolating cytotoxin a (VacA)

As mentioned above, the higher vacuolation activity of strains carrying *vacA* s1, i1 or m1 genotypes were linked with more severe clinical outcomes than the s2, i2 or m2 genotypes [20, 32, 33, 36, 58, 59]. However, similar to *cagA* genoprevalence, diversity in the prevalence of *vacA* risky genotypes (s1, i1 and m1) was noted in different geographic regions. Therefore, the use of *vacA* genotypes to determine PUD risk depends on the prevalence of risky genotypes in each geographic region. In America, Europe, Africa and Middle East where the prevalence of *vacA* risky genotypes (s1/m1) is lower, individuals infected with *vacA* s1 or m1 *H. pylori* strains have an increased risk of PUD compared with those with s2 or m2 strains [20, 32, 36, 59]. In Southeast Asia, *vacA* m1 is associated with increased risk of PUD [58]. In East Asia, where most strains are *vacA* s1/i1/s1 genotype, *vacA* genotypes cannot differentiate high risk infectors, and other virulence markers should be used [35]. The *vacA* i1 genotype is not associated with risk of PUD in meta-analysis [34].

### Blood group antigen binding adhesin (BabA)

Both animal and human studies showed that infection by BabA-expressing strains is associated with higher bacterial density and more severe injury in the gastric mucosa [46, 60]. A meta-analysis of case-control studies showed that *babA2* genopositive is associated with an increased risk of PUD (OR = 2.07) in Western countries, but not in Asian countries [45]. As mentioned above, Fujimoto et al. determined BabA expression level by immunoblotting [46]. BabA-L (BabA low producers) and BabA-H (BabA high producers) strains were also associated with higher risk of duodenal ulcer than BabA-negative strains in Western countries (OR = 33.9 and 18.2, respectively) [46]. However, the underlying mechanisms remained to be elucidated. Despite the positive findings of *babA2* genopositive and BabA expression intensity in determining peptic ulcer risk in Western countries, these methods are not sensitive enough to differentiate high risk infector in East Asia. Further studies using multiple sets of *babA* and *babB* PCR primers [44] are warranted to determine whether *babA/B* recombination can determine ulcer risk.

### Duodenal ulcer promoting gene (DupA)

DupA was initially identified in 2005 and named for its role to increase risk of DU (i.e. duodenal ulcer promoting). The original data showed the presence of *dupA* gene was associated with increased risk of DU, as well as neutrophil infiltration and IL-8 expression in the antrum [61]. In contrast, its presence was also associated with decreased risk of gastric atrophy, intestinal metaplasia, and gastric cancer [61]. The data are compatible with the findings that antral predominant gastritis often leads to higher gastric acid secretion and duodenal ulcer formation. Nevertheless, although two meta-analyses found a small increase in DU risk (OR = 1.4) by *dupA*-genopositive strain [62, 63], conflicting results were found [64, 65]. In addition, the association was only found in Asian countries, but not in Western countries [63]. It has been reported that *dupA* forms T4SS with *vir* genes around it (called *dupA* cluster). *H. pylori* with complete *dupA* cluster was associated with 2.1-fold risk of DU than that with incomplete *dupA* cluster or negative *dupA* [66]. Therefore, merely testing the presence/absence of *dupA* gene may not reflect the competent function of DupA and analysis of whole *dupA* cluster may be more accurate to determine DU risk, especially in Western countries [67].

### Induced by contact with epithelium gene a (IceA)

The *iceA* gene was originally identified in 1998 when investigating genes “**i**nduced by **c**ontact of *H. pylori* with **e**pithelium” [68]. Two families of *iceA* have been found, *iceA1* and *iceA2*. Infection with *iceA1-*genopositive strain is associated with PUD and increased mucosal levels of IL-8 [57, 68, 69]. Meta-analysis showed the presence of *iceA1* gene was associated with a small increase of peptic ulcer risk (OR  = 1.28) in Western countries [70].

### Outer inflammatory protein (OipA)

OipA is an outer membrane protein that functions in adhesion and IL-8 induction. Its functional status (on or off) is regulated by slipped-strand mispairing based on the number of CT dinucleotide repeats in the 5′ region of *oipA* gene [71]. Infection with *oipA* “on” strain has been linked with higher *H. pylori* colonization density, neutrophil infiltration and IL-8 levels in the human stomach [72]. However, the corresponding receptor for OipA has not been identified.

Previous study showed *oipA* “on” status was closely linked to *cagA-*positive, *vacA* s1/m1, and *babA2-*positive genotypes [73]. Moreover, *oipA* “on” status was associated with increased risk of DU independent of the other virulence factors [72]. A meta-analysis also reported that the *oipA* “on”, but not “off”, status is significantly associated with an increased risk of PUD (OR = 3.97) and gastric cancer (OR = 2.43), especially in the Western countries [74]. Importantly, merely investigating the presence/absence of *oipA* gene would overlook its functional on/off status and may be unreliable to predict risks of PUD or GC [74].

## Conclusions

Epidemiological studies had provide evidence that infection with *H. pylori* carrying specific virulence factors is associated with increased risk of serious clinical outcomes. To identify infectors who are at high risk of serious clinical outcomes, one should select appropriate virulence factors and testing methods according to the epidemiological data of each geographic area and ethnic group.

## Abbreviations

*cag* PAI*c*: *ag* pathogenicity island
CagA: Cytotoxin-associated gene A
DU: Duodenal ulcer
EPIYA: Glu-Pro-Ile-Tyr-Ala
ERK 1 and ERK 2: Endoplasmic reticulum kinases 1 and 2
GGT: Gamma-glutamyl transpeptidase
GU: Gastric ulcer
HtrA: High temperature requirement A
LPS: Lipopolysaccharide
MALToma: B cell lymphoma of the mucosa associated lymphoid tissue
MGMT: O6-methylguanine DNA methyltransferase
PUD: Peptic ulcer disease
RUNX3: Runt-related transcription factor 3
SHP-2: Src homology 2 phosphatase
T4SS: Type IV secretion system
TFF2: Trefoil factor 2
VacA: Vacuolating cytotoxin A
